# The Impact of Intensive Care Unit Patient Care on Hostility Levels Among Relatives

**DOI:** 10.1192/j.eurpsy.2025.1922

**Published:** 2025-08-26

**Authors:** Z. C. Konstanti, S. Kotrotsiou, M. Kourakos, S. Mantzoukas, E. Kotrotsiou, V. Koulouras, M. Gouva

**Affiliations:** 1 DEPARTMENT OF NURSING, UNIVERSITY OF IOANNINA RESEARCH LABORATORY OF PHYCHOLOGY OF PATIENTS, FAMILIES AND HEALTH PROFESSIONALS, SCHOOL OF HEALTH SCIENCES, IOANNINA; 2 DEPARTMENT OF NURSING, UNIVERSITY OF PATRA, PATRA; 3 DEPARTMENT OF NURSING, UNIVERSITY OF IOANNINA RESEARCH LABORATORY OF INTEGRATED HEALTH, CARE AND WELL BEING, SCHOOL OF HEALTH SCIENCES, IOANNINA, Greece; 4 DEPARTMENT OF NURSING, FREDERICK UNIVERSITY, NICOSIA, Cyprus; 5 DEPARTMENT OF ICU, MEDICINE SCHOOL, UNIVERSITY OF IOANNINA, IOANNINA, Greece

## Abstract

**Introduction:**

Hostility is defined as an enduring tendency in individuals to perceive the opinions and actions of others as negative or to anticipate aggression. This study examines how the Intensive Care Unit (ICU) admission of a critically ill family member influences the hostility levels of their relatives.

**Objectives:**

This study aims to assess the hostility levels of family members of ICU patients, focusing on their sociodemographic characteristics and the closeness of their relationship to the patient.

**Methods:**

The study included first-degree relatives, close relatives, and intimate friends of ICU patients. Data were collected via written questionnaires completed by relatives during the first week of the patient’s ICU stay. Instruments used were a sociodemographic questionnaire and the Hostility and Direction of Hostility Questionnaire (HDHQ).

**Results:**

A total of 223 family members (mean age: 41.5 ± 11.9 years), representing 147 critically ill patients, participated. Among the participants, 81 (36.3%) were male and 142 (63.7%) were female. The majority were the patients’ children (40.8%), siblings (19.3%), or companions (16.1%). The mean hostility scores for male and female relatives were 20.54 ± 8.16 and 19.36 ± 7.43, respectively, with no statistically significant difference (P = 0.271). However, a statistically significant difference (P = 0.030) was found between relatives living in the same household as the patient (21.38 ± 8.47) and those living separately (18.92 ± 7.13).

**Conclusions:**

This study highlights elevated hostility levels among relatives of ICU patients, suggesting that hostility may fluctuate in response to external stressors such as the critical illness of a loved one. The findings support the notion that hostility, while a personality trait, may also function as an adaptive behavior influenced by situational challenges, such as those faced when a family member is admitted to intensive care.

**Disclosure of Interest:**

None Declared

